# Correlation between Buruli Ulcer Incidence and Vectorborne Diseases, Southeastern Australia, 2000–2020

**DOI:** 10.3201/eid2712.203182

**Published:** 2021-12

**Authors:** Jake Andrew Linke, Eugene Athan, N. Deborah Friedman

**Affiliations:** Deakin University School of Medicine, Geelong, Victoria, Australia (J.A. Linke, E. Athan);; Barwon Health, Geelong (E. Athan, N.D. Friedman)

**Keywords:** Buruli ulcer, Mycobacterium ulcerans, transmission, bacteria, vector-borne infections, Australia

## Abstract

Researchers have hypothesized that mosquitoes are vectors involved in *Mycobacterium ulcerans* transmission. Previous findings of a correlation between incidence of *M. ulcerans*, which causes Buruli ulcer, and locally acquired vectorborne diseases in southeastern Australia further strengthened this argument. However, our updated data indicate that this correlation has not continued beyond 2008.

*Mycobacterium ulcerans* infection causes skin and soft tissue destruction and is classified by the World Health Organization as a neglected tropical disease. Internationally, disease caused by *M. ulcerans* infection is known as Buruli ulcer (BU) and has been reported in 33 countries (*1*), mostly in central and western Africa. However, in southeastern Australia, the epidemic is worsening; disease incidence and severity of infection have increased rapidly since 2015 (*2*). Although the mode(s) of transmission of *M. ulcerans* remain(s) unclear, a study published in 2009 showed a correlation between BU incidence and locally acquired vectorborne diseases in southeastern Australia (*3*). This finding strengthened the hypothesis that mosquitoes may be involved in *M. ulcerans* transmission. We examined data to determine if this correlation continued beyond 2008.

In Africa, *M. ulcerans* is thought to be transmitted by water bugs, and a report of a case in southeastern Australia suggested that a BU lesion first appeared at the site of a mosquito bite (*4*). Subsequently, the probability of mosquitoes being *M. ulcerans* positive by PCR has been associated with the degree of BU endemicity in southeastern Australia (*4*,*5*). Furthermore, being bitten by a mosquito substantially increases the odds of BU developing, and using insect repellent and protective clothing reduces the odds (*6*). However, this association does not necessarily imply that mosquitoes are involved in *M. ulcerans* transmission because covering limbs with clothing would also help to protect against other potential environmental sources of *M. ulcerans*, such as possum excreta or soil contamination of wounds. Furthermore, mosquitoes rarely act as vectors for bacteria, and no other species of *Mycobacteria* are known to have arthropod vectors.

The apparent role of mosquitoes in the transmission of *M. ulcerans* may be explained by mechanical vectoring. Within *M. ulcerans–*endemic areas of southeastern Australia, the geographic locations of BU cases are highly focal. Cases are often clustered together, and adjoining communities only a few kilometers away may be spared (*7*). However, *Aedes camptorhynchus* mosquitoes, one of the main species thought to be involved in *M. ulcerans* transmission, are widespread within *M. ulcerans*–endemic and –nonendemic areas, and many species of mosquito may have the capacity to fly distances that would take them outside of BU-affected regions. Therefore, the transmission model for *M. ulcerans* may not be explained by mosquitoes alone.

In coastal regions of southeastern Australia, Ross River virus (RRV) and Barmah Forest virus (BFV) cause locally acquired vectorborne diseases. RRV and BFV are transmitted by *Ae. camptorhynchus* mosquitoes, which were the main species captured during an *M. ulcerans* outbreak in Point Lonsdale, southeastern Australia (*4*). The incidence of RRV and BFV peaks sporadically, especially during years of above average rainfall or La Niña events, as occurred during the 2020–21 summer in Australia (*8*). These environmental changes often favor increased mosquito population sizes, thus giving rise to RRV and BFV outbreaks (*9*). It is also thought that BU incidence is associated with environmental factors; increased incidence has lagged 12 months behind periods of greater rainfall (*10*). However, no association has been found between rainfall and BU cases on the Mornington Peninsula, the main *M. ulcerans*–endemic site driving the increased incidence of BU in southeastern Australia.

During 2002–2008, BU incidence correlated with combined RRV/BFV incidence in Victoria, southeastern Australia (r^2^ = 0.52) (*3*). It was argued that this correlation strengthened the link between mosquitoes and *M. ulcerans* transmission in southeastern Australia. However, this observation was made over a short time. Using the square of the Pearson product-moment correlation coefficient (coefficient of determination) analysis over a 21-year period (2000–2020), we found little to no correlation between BU and combined RRV/BFV incidence in southeastern Australia (r^2^ = 0.05; p = 0.69) (Figure) (Appendix). For comparison, during this same period there was no correlation between BU and infection with *M. tuberculosis* (the other main mycobacterial disease in southeastern Australia) or *Legionella* (water-associated bacteria).

**Figure:**
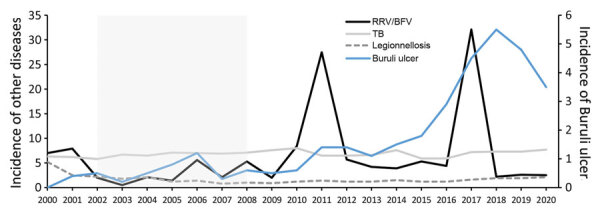
Incidence (cases/100,000 population) of Buruli ulcer compared with that of other notifiable diseases in Victoria, Australia, during 2000–2020. Victoria is located in southeastern Australia. “Other diseases" on left y-axis indicates TB, legionellosis, and RRV and BFV incidence combined. The shaded area (2002–2008) denotes a period when Buruli ulcer incidence correlated with RRV/BFV incidence (*3*). In Australia, these infections are notifiable and incidence rates are publicly available (*8*). BFV, Barmah Forest; RRV, Ross River virus; TB, tuberculosis.

A lack of correlation between BU incidence and locally acquired vectorborne diseases does not disprove that mosquitoes are involved in *M. ulcerans* transmission. Nevertheless, this lack of correlation may suggest that the worsening BU epidemic in southeastern Australia is not caused by increased mosquito populations or other environmental changes that favor RRV and BFV outbreaks. We believe that other independent factors may be driving the increased BU incidence, although the effects of recent La Niña events on BU incidence in 2021 are not yet known. Planning and implementing successful public health interventions to control *M. ulcerans* are substantially hindered by lack of knowledge of the mechanism of disease transmission. 

Appendix Additional information for study of correlation between incidence of Buruli ulcer and vectorborne diseases in southeastern Australia, 2000–2020.
