# Supplementary material for: Correlation between Buruli Ulcer Incidence and Vectorborne Diseases, Southeastern Australia, 2000–2020
Source: Emerg Infect Dis. 2021 Dec;27(12):3191–2. doi: 10.3201/eid2712.203182 (PMC8632172; doi:10.3201/eid2712.203182)
Supplement: Appendix — Additional information for study of correlation between incidence of Buruli ulcer and vectorborne diseases in southeastern Australia, 2000–2020. [file 20-3182-Techapp-s1.pdf]

Article DOI: https://doi.org/10.3201/eid2712.203182

Correlation between Buruli Ulcer Incidence and Vectorborne Diseases, Southeastern Australia, 2000–2020

Appendix

Case Definition

Buruli ulcer is a microbiological diagnosis defined as a patient presenting with clinical features suggestive of disease, who inhabits or has visited an endemic region, and tests positive for *M. ulcerans* by PCR or culture from either a swab or tissue biopsy. Although disease severity may differ for each patient, a microbiological specimen and diagnosis is only sought if a patient has features suggestive of BU. This means that the reported incidence of BU and *M. ulcerans* is the same and they can be used interchangeably when referring to reported incidence, for example *Mycobacterium ulcerans* (Buruli ulcer) incidence.

Surveillance

Medical practitioners and laboratories in the state of Victoria, located in southeastern Australia, are required by law to notify the Victorian Department of Health of positive *Mycobacterium ulcerans* (Buruli ulcer) cases. The Victorian Department of Health collates this information and then publically releases detailed data on incidence (Department of Health and Human Services Victoria 2021).

Statistical Analysis

Incidence data was transferred to SPSS V.27 software for analysis. The square of the Pearson product-moment correlation coefficient (also known as coefficient of determination or Pearson R) was used to calculate r^2. This is the same method used by Johnson and Lavender to calculate correlation from 2002–2008.

Appendix Table. Rate per 100,000 of selected notifiable diseases in Victoria, Australia, from 2000–2020 (Department of Health and Human Services Victoria 2021).

Year	Buruli ulcer	(Ross River virus/ Barmah Forest virus)	TB	Legionellosis
2020	3.5	2.5	7.7	2.1
2019	4.8	2.6	7.3	1.9
2018	5.5	2.2	7.3	1.9
2017	4.5	32.1	7.2	1.6
2016	2.9	4.4	5.9	1.2
2015	1.8	5.3	5.9	1.2
2014	1.5	3.9	7.6	1.5
2013	1.1	4.2	6.6	1.2
2012	1.4	5.7	6.5	1.2
2011	1.4	27.5	6.5	1.4
2010	0.6	8.4	8	1.2
2009	0.5	2	7.6	0.9
2008	0.6	5.3	7.1	1
2007	0.3	2.1	6.9	0.8
2006	1.2	5.6	7	1.4
2005	0.8	1.4	7.1	1.2
2004	0.5	2.1	6.5	2.1
2003	0.2	0.5	6.7	1.8
2002	0.5	2	5.8	2.2
2001	0.4	7.9	6.2	2.5
2000	0	7	6.3	5.2
